# Nurses' Trials—Weariness

**Published:** 1887-02-05

**Authors:** 


### Words of Consolation.

[Communications for this column should be addressed, "Chaplain," care of the Editor of The Hospital, who will gratefully welcome any suggestions or inquiries from matrons, nurses, and the staff generally.]

### XIX.—Nurses' Trials—Weariness.

Weariness, both of mind and body, must be one of the severest trials to which nurses are subject. The office of nurse is seldom undertaken permanently until a woman's physical strength has been thoroughly tested. But assuming that a nurse's health is all that can be desired, she cannot expect to escape the heavy burden of weariness; for weariness is almost inseparable from watchfulness. Let alone the question of hard work, irregular hours, or very intermittent sleep, the mind which has to sacrifice itself continually in efforts of watchfulness, must needs grow weary as well as the body. Our Lord's example in this matter is full of consolatory thoughts. He never suffered, so far as we know, from any sickness. His health was probably very perfect. For, being miraculously born, He was not subject to any hereditary tendencies to disease; nor do we ever read of His suffering from anything of the nature of an inherent indisposition. But His public life, at least, was occupied with incessant goings to and fro. He lived, as we should say, at high pressure, with only intermittent sleep, with often nowhere to lay His head, irregular meals, fastings, long journeys on foot, not to mention the frequent going forth from His person of the virtue of healing power. All these things must have contributed to weariness and exhaustion in a marked degree. "Come ye yourselves into a desert place and rest awhile" (Mark vi. 31), was not spoken only for the disciples, as if the Master had no desire to share the retreat, or had no interest in gaining refreshment for Himself. They are rather the words of One who was very weary Himself, and craved for rest. It was after one of His hardest days that He was thankful to take advantage even of a rough voyage, and slept soundly while the waves were breaking over the ship. The physically strong have much to go through, if only because so much is expected of them; and because they are known to be strong, they are not supposed to feel the pain of weariness to any great extent. We doubt this. We believe that weariness is one of their heaviest crosses. And yet there comes along with such a trial, sometimes, the most intense satisfaction at the thought of their labour not having been in vain in the Lord. There were periods like this in our Lord's suffering life. He rejoiced in spirit. Surely such rejoicing was sometimes over having spent Himself in such a noble cause. He could look, too, upon every detail of His work as highly finished. The very pain arising from watchfulness was not without its glory. We may be too well satisfied with our labour, when it has been very imperfectly performed. But we are not concerned at this moment with temptations to vain glory. Rather, we would console weary watchers, by night or by day, by recalling the satisfaction there is to be found in weariness begotten in a noble cause. Yet even in the midst of such a trial, if we can only believe that Christ has been present in us, and that the expenditure of power from our own system has been the going forth of His virtue towards the weaker members of His body. Ah, then what gratitude may not spring up in our hearts at being privileged as instruments for the dispensing of Divine energy. The power came from Him, and was used for Him. The seeming waste experienced in mind and body has left us as He was in the world. Is there no glory in such weariness as this? Yes, certainly there is. The more so if it has been your lot to reflect that your high calling is a constant preservation against a selfish expenditure of energy. Think of the multitudes of people in the world wearying themselves to death over selfish sordid objects without a thought of benefitting any but themselves. Think how these are ever breeding in themselves a weariness they must bitterly repent of or continue to suffer for. Set their lot beside your own; and let the comparison urge you to a more thorough consecration of your labour to Christ day by day. Then, as often as a feeling of weariness creeps over you, even though you have hours to wait before you can rest, you may offer your very exhaustion to the Father in sure and certain hope of its acceptance.

(*To be continued.*)

